# Genome-wide identification of accessible chromatin regions in bumblebee by ATAC-seq

**DOI:** 10.1038/s41597-020-00713-w

**Published:** 2020-10-26

**Authors:** Xiaomeng Zhao, Long Su, Weilin Xu, Sarah Schaack, Cheng Sun

**Affiliations:** 1 Institute of Apicultural Research, Chinese Academy of Agricultural Sciences, Beijing, China; 2 Department of Biology, Reed College, Portland, OR USA

**Keywords:** Entomology, Gene regulation, Chromatin, Sequencing

## Abstract

Bumblebees (Hymenoptera: Apidae) are important pollinating insects that play pivotal roles in crop production and natural ecosystem services. Although protein-coding genes in bumblebees have been extensively annotated, regulatory sequences of the genome, such as promoters and enhancers, have been poorly annotated. To achieve a comprehensive profile of accessible chromatin regions and provide clues for all possible regulatory elements in the bumblebee genome, we performed ATAC-seq (Assay for Transposase-Accessible Chromatin with high-throughput sequencing) on *Bombus terrestris* samples derived from four developmental stages: egg, larva, pupa, and adult, respectively. The ATAC-seq reads were mapped to the *B. terrestris* reference genome, and its accessible chromatin regions were identified and characterized using bioinformatic methods. We identified 36,390 chromatin accessible regions in total, including both shared and stage-specific chromatin accessible signals. Our study will provide an important resource, not only for uncovering regulatory elements in the bumblebee genome, but also for expanding our understanding of bumblebee biology throughout development.

## Abstract (summary)

**Table:** 

Measurement(s)	open_chromatin_region
Technology Type(s)	ATAC-seq
Factor Type(s)	developmental stage
Sample Characteristic - Organism	Bombus terrestris

Machine-accessible metadata file describing the reported data: 10.6084/m9.figshare.13047905

## Background & Summary

Bumblebees (Hymenoptera: Apidae) are important pollinating insects that play pivotal roles in crop production and natural ecosystem services^1,2^. They were well known as excellent pollinators of greenhouse tomato crops, decreasing the cost of labour and improving the yield and quality of fruit^1,3^. Now, their usage has been expanded to other crops, including those grown in poly-tunnels and open fields^4^. Also, bumblebees are ecologically important pollinators, with a large number of wild plants being pollinated predominantly or exclusively by bumblebees^1,5^. Because they are holometabolous insects that undergo four developmental stages (egg, larva, pupa, adult), they are also useful models to study mechanisms underlying developmental signalling and plasticity^6^. Regulatory elements play a major role in controlling the temporal and spatial expression of genes, through which they control the development and physiology of an organism^7^. To date, the protein-coding sequences of bumblebees have been extensively annotated^8,9^. However, regulatory elements, such as promoters, enhancers, and silencers, have been poorly annotated in the bumblebee genome.

ATAC-seq (Assay for Transposase-Accessible Chromatin with high-throughput sequencing) is a fast and highly-sensitive method that can determine accessible chromatin regions across the genome^10,11^, from which regulatory sequences can be inferred genome-wide. This technique not only requires less starting material, but also produces more precise results than previous approaches^12,13^. Furthermore, ATAC-seq can detect chromatin accessibility using whole animal preparations (containing mixtures of tissues or organs) with high sensitivity^14^.

**Fig. 1:**
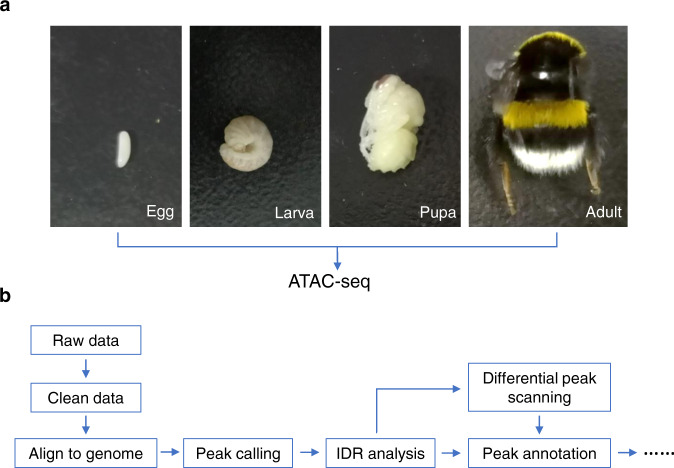
An overview of study design and data analysis workflow. (**a**) The four developmental stages of bumblebee used for ATAC-seq. (**b**) The analysis workflow of ATAC-seq data.

In this study, we used ATAC-seq to perform a genome-wide survey of accessible chromatin regions in *Bombus terrestris*, the most widely used commercial bumblebee species globally^3^. To achieve a comprehensive profile of open chromatin regions and provide clues for all possible regulatory elements in the bumblebee genome, we generated eight chromatin accessibility datasets for *B. terrestris* samples derived from its four developmental stages: egg, larva, pupa, and adult, respectively, with two biological replicates for each stage (Fig. 1a). In this experiment, we used whole animals, containing multiple cell types, for ATAC-seq. Therefore, our approach generates an atlas of open chromatin in each developmental stage, which does not necessarily mean that the focal chromatin region is open in each cell type. Our integrative ATAC-seq bioinformatic analysis workflow is shown in Fig. 1b. The accessible chromatin regions identified by this study will provide important resources for uncovering promoters, enhancers and other regulatory elements in the bumblebee genome. A total of 5,694, 4,850, 13,126 and 12,720 chromatin accessible regions were identified for developmental stage of egg, larva, pupa, and adult, respectively (Table 1).

**Table 1:** The summary of ATAC-seq, read mapping and peak calling results.

Developmental stage	Sample ID	Raw reads	Clean reads	Mapped reads	Candidate peaks	IDR peaks	Accession #
Egg	E1	108,102,554	107,917,012	88,127,516	33,612	5,694	GSM4592423
	E2	108,096,978	107,593,940	81,946,129	30,268		GSM4592424
Larva	L1	108,104,322	107,689,026	68,377,504	17,073	4,850	GSM4592421
	L2	108,085,350	107,755,740	97,600,443	12,331		GSM4592422
Pupa	P1	108,103,224	107,529,074	86,046,510	33,149	13,126	GSM4592419
	P2	108,099,388	107,345,156	93,595,048	37,502		GSM4592420
Adult	A1	108,101,410	107,911,474	65,330,095	27,135	12,720	GSM4592417
	A2	108,096,400	107,671,438	87,663,901	31,548		GSM4592418

*IDR peaks: peaks passing IDR cutoff of 0.05.

To understand the position of accessible chromatin regions relative to their nearest gene, we compared the coordinates of ATAC-seq peaks with that of *B. terrestris* protein-coding genes. Using this approach, if we consider 2 kb upstream of TSS putative promoter regions, at least 35% of accessible chromatin regions are located in promoter regions. Open chromatin regions could also be found in exons, introns, and distal intergenic regions (Fig. 3b–e). Enhancers could be found far from their target genes or even within exons and introns^15,16^, thus the identified open chromatin in such regions represents the best candidates for enhancers in the bumblebee genome. In addition, the information obtained will expand our understanding of bumblebee biology, generally, and facilitate the cloning of bumblebee genes that control important traits.

The accessibility of regulatory elements is crucial and strongly associated with gene transcriptional activity, which reveals real-time gene expression during developmental and physiological processes^17^. Differences in accessible chromatin signals among different developmental stages could lay the foundation for further metamorphosis research. We deposited the identified differential ATAC-seq peaks between developmental stages in Figshare^18^.

## Methods

### Sample collection

Commercial *B. terrestris* colonies were bought from Koppert China (http://www.koppert.cn). Worker bee samples were collected from each of the four developmental stages: egg, larva, pupa, and adult, respectively, with two biological replicates for each stage (as listed in Table 1). Each sample was comprised of either an individual adult worker bee or pooled tissue of another developmental stage (egg, whole larva or whole pupa) equivalent to the volume of an adult worker bee. The eggs we collected were straight and smooth; all larvae had a C-shape curve; pupae had visible compound eye pigmentation and clear head-thorax-abdomen segmentation, but their wings were not developed yet; adult bees were bright with dense hair, and could flap their wings (Fig. 1a). All samples were frozen in liquid nitrogen after collection.

### ATAC-seq protocol

ATAC-seq was performed by BGI-Shenzhen (https://en.genomics.cn), following previously published protocols^10,11^. Briefly, about 50,000 intact and homogenous cells were harvested and counted for each developmental stage, which were then centrifuged for 5 min at 500 × g, 4 °C. After discarding supernatant, the pellet was gently re-suspended with 50 µL of cold 1x PBS buffer, followed by 5 min of centrifuging at 500 × g, 4 °C. After removing supernatant, the pellet was gently pipetted and resuspended in 50 µL of cold lysis buffer (10 mM Tris-HCl, pH 7.4, 10 mM NaCl, 3 mM MgCl2, 0.1% IGEPAL CA-630) to release nuclei. After lysis, the suspension was spun at 500 × g for 10 minutes, 4 °C. After centrifugation, the pellet was immediately resuspended in the transposase reaction mix (25 μL 2x TD buffer, 2.5 μL Transposase (Illumina) and 22.5 μL of nuclease free water. The purified products were amplified in a 50 µL of reaction mixture containing the purified transposed DNA, 1x NEBnext High-Fidelity PCR master mix and 1.25 μM of custom Nextera PCR primers, with the following PCR program: (1) 72 °C, 5 minutes; (2) 98 °C, 30 seconds; (3) 98 °C, 10 seconds; (4) 63 °C, 30 seconds; (5) 72 °C, 1 minute; (6) Repeat steps 3–5 for 4 times; (7) Hold at 4 °C. After amplification, the PCR products obtained were purified by Qiagen MinElute PCR Purification Kit, with the purified PCR products being eluted in 20 µL Elution Buffer (10 mM Tris Buffer, pH 8). Next, the purified PCR products were used to produce single-strand DNA circles, from which DNA nanoballs were generated by rolling circle replication as previously described^19^. Finally, the DNA nanoballs were sequenced on the BGISEQ-500 sequencing platform, generating paired-end reads with a read length of 50 bp.

### ATAC-seq data analysis

Raw reads were filtered first to remove low-quality reads and adaptor sequences by SOAPnuke^20^. Clean reads were mapped to the reference genome of *B. terrestris* (GenBank: GCF_000214255.1) using Bowtie2^21^. The fragment length distribution of ATAC-seq was determined by the “fragSizeDist” function of R package ATACseqQC (Version: 1.12.3)^22^. The read coverages for genomic regions were computed and genome-wide similarities were assessed between the two replicates of the same developmental stage using deepTools^23^, with “multiBamSummary” and “plotCorrelation” function. We used MACS2 to call peaks (open chromatin regions) with parameters as reported previously^14,24,25^. The Irreproducible Discovery Rate (IDR) analysis was used to evaluate the reproducibility of high-throughput experiments by measuring consistency between the two biological replicates of the same developmental stage^26^. Peaks passing the suggested threshold (IDR < = 0.05) were reproducible peaks between the two replicates, which were retained for further analyses. The frequencies of peaks around transcription start sites (TSSs) were plotted by the “plotAvgProf” function of R package Chipseeker (Version: 1.24.0)^27^ based on *B. terretris* genome annotation file (Bter_1.0.46.gff3.gz on http://metazoa.ensembl.org/Bombus_terrestris/Info/Index). DEseq2 was employed to identify differential peaks between developmental stages using R package DiffBind (Version: 2.16.0)^28^. Peaks with FDR < 0.05 were treated as differential peaks between each two developmental stages. To retrieve the nearest genes around reproducible peaks of each developmental stage and differential peaks between different developmental stages, the coordinates of peaks were compared with the annotation of *B. terrestris* genome with the following priority order: promoter (−2kb, TSS), 5′UTR, 3′UTR, exon, intron, downstream (TES, 3 kb) and distal intergenic region. The distribution of accessible regions was plotted in pie charts by ChIPseeker^27^.

## Data Records

Accession numbers are listed in Table 1. All ATAC-seq reads and peak files have been submitted to the NCBI Gene Expression Omnibus (NCBI GEO)^29^, which are accessible through GEO Series accession number GSE151858^30^. The sequencing data for every developmental stage (in fastq format) have been linked to the Sequence Read Archive of NCBI under the accession number SRP266094^31^. Reproducible peaks of each developmental stage, as well as differential peaks between developmental stages, have been deposited in Figshare^18^.

## Technical Validation

**Fig. 2:**
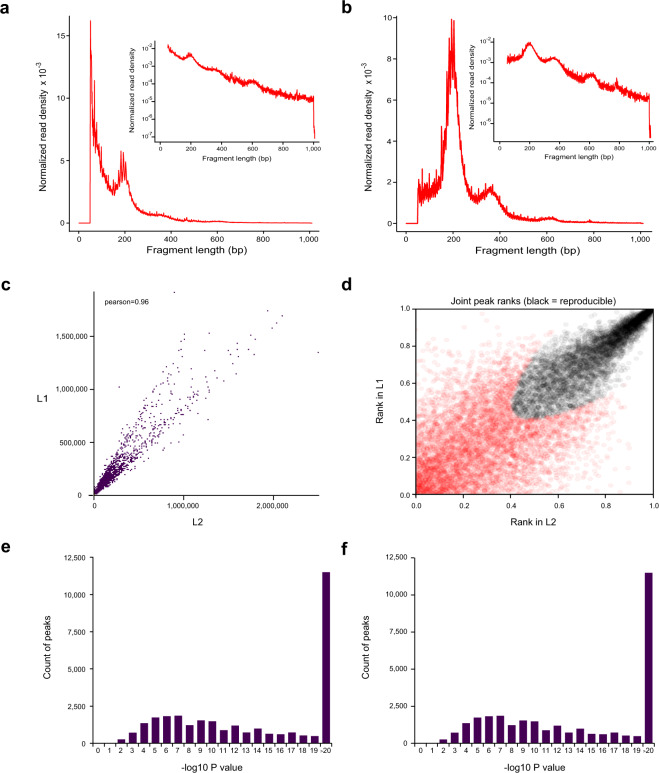
The quality metrics of ATAC-seq data. (**a**,**b**) Fragment size distribution of ATAC-seq reads for developmental stage larva (sample ID: L1 and L2, respectively). (**c**) Pearson correlation of the two biological replicates of stage larva. (**d**) IDR analysis of ATAC-seq peaks obtained from stage larva. The scatter plot shows one point for every peak, with its location representing in rank in each replicate. Peaks that pass the specified IDR threshold are coloured in black. (**e**,**f**) Peak score (-log10 (P value)) distribution for sample L1 (**e**) and L2 (**f**), respectively.

**Fig. 3:**
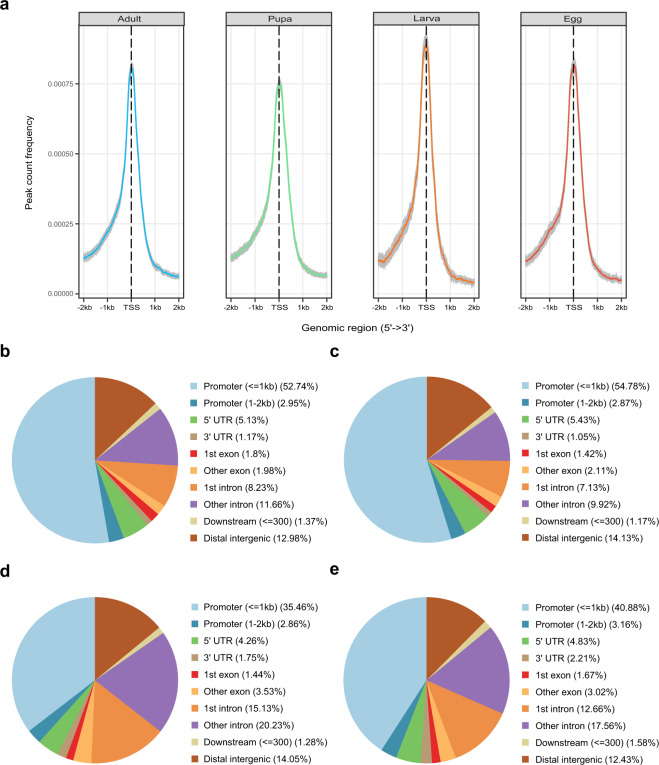
Features of ATAC-seq data in bumblebee. (**a**) The profile of chromatin accessible signals around bumblebee genes for each developmental stage. TSS represents transcription start site. (**b**–**e**) Reproducible peaks from egg, larva, pupa and adult stage, respectively, were annotated and classified based on their relative locations to nearest genes.

Raw reads of ATAC-seq data were first filtered to remove adaptor sequences, contamination and low-quality reads. Clean reads were mapped to the reference genome of *B. terrestris* (Table 1). Based on the mapping results, we inferred the fragment size distribution. As expected, while a majority of fragments were shorter than one nucleosome length (approximately 150 bp), there were also significant number of fragments longer than this length and displayed periodicity (Fig. 2a,b; figures in Figshare^18^). Pearson correlation analysis was used to calculate and visualize pairwise correlation values between the two replicates of the same developmental stage, and results showed that correlation coefficients of each group were all greater than 0.95 (Fig. 2c; figure in Figshare^18^). Peaks (potential accessible chromatin regions) were called by MACS2 for each replicate of the four developmental stages. Most peaks are with peak score (−log10 (P value)) > 20 (Fig. 2e,f; figure in Figshare^18^), indicating the high reliability of peak calling. IDR method was applied to find reproducible peaks between replicates of the same developmental stage (Table 1; Fig. 2d; figure in Figshare^18^), and on average, 45% of peaks could pass the threshold (IDR < = 0.05) for each developmental stage. The intensity of ATAC-seq signal corresponds to the level of chromatin accessibility and can be used to identify poised and active regulatory regions genome-wide. We plotted chromatin accessible signals around genes for each developmental stage (using reproducible peaks for each stage), and as expected, the regions around transcription start sites were enriched for these signals (Fig. 3a). Open chromatin regions could also be found in exons, introns, and distal intergenic regions (Fig. 3b–e), therefore, except for protomers, our ATAC-seq dataset could also be used to identify other types of regulatory elements in bumblebee genome^32^. Peaks obtained from each developmental stage were used to identify differential open chromatin sites among the four developmental stages. Only peaks with FDR < 0.05 (based on DEseq2 method) were treated as differential sites, which can be used for further analysis of metamorphosis in bumblebees.

Bumblebees in different developmental stages exhibit differential morphological features and behave differently due to the temporal and spatial regulation of gene expression. We used ATAC-seq to perform a genome-wide survey of accessible chromatin regions in bumblebee (*B. terrestris*) by integrating data obtained from its four distinct developmental stages. The identified open chromatin regions will provide resources to uncover candidate regulatory sequences, such as promoters, enhancers and other *cis*-regulatory elements in the bumblebee genome. Also, the identified differential ATAC-seq peaks between developmental stages will be useful to identify genes or pathways involved in insect metamorphosis.

## Data Availability

SOAPnuke: Version: 2.1.2. Parameters: filter -l 5 -q 0.5 -n 0.1 -Q 2 –5 1 -c 50. Bowtie2: Version: 2.2.5. Parameters: -q --phred64 --sensitive --dpad 0 --gbar 99999999 --mp 1,1 --np 1 --score-min L,0, -0.1 -I 1 -X 1000 -p 16 -k 200.deepTools: Version: 3.4.3. Parameters: --corMethod pearson --whatToPlot scatterplot --skipZeros --removeOutliers.MACS2: Version:2.2.5. Parameters: --nomodel --extsize 200 --shift -100 -- format BAM --gsize 2.17e8 -- call-summits.IDR: Version: 2.0.3. Parameters: --input-file-type narrowPeak --rank p.value --plot --log-output-file.
